# The role of EMT-related lncRNA in the process of triple-negative breast cancer metastasis

**DOI:** 10.1042/BSR20203121

**Published:** 2021-02-03

**Authors:** Haomeng Zhang, Jiao Wang, Yulong Yin, Qingjie Meng, Yonggang Lyu

**Affiliations:** 1 Department of Thyroid and Breast Surgery, Xi’an No. 3 Hospital, The Affiliated Hospital of Northwest University, Fengcheng 3rd Road 10, Weiyang District, Xi’an, Shaanxi 710018, P. R. China; 2 Department of Radiotherapy, The First Affiliated Hospital of Xi'an Jiaotong University, Yanta West Road 277, Yanta District, Xi’an, Shaanxi 710061, P. R. China

**Keywords:** Triple-negative breast cancer(TNBC), long non-coding RNA(LncRNA), Metastasis, Epithelial-mesenchymal transition(EMT)

## Abstract

Triple-negative breast cancer (TNBC) is the most malignant and fatal subtype of breast cancer, which has characterized by negativity expression of ER, PR, and HER2. Metastasis is the main factor affecting the prognosis of TNBC, and the process of metastasis is related to abnormal activation of epithelial–mesenchymal transition (EMT). Recent studies have shown that long non-coding RNA (LncRNA) plays an important role in regulating the metastasis and invasion of TNBC. Therefore, based on the metastasis-related EMT signaling pathway, great efforts have confirmed that LncRNA is involved in the molecular mechanism of TNBC metastasis, which will provide new strategies to improve the treatment and prognosis of TNBC. In this review, we summarized many signal pathways related to EMT involved in the transfer process. The advances from the most recent studies of lncRNAs in the EMT-related signal pathways of TNBC metastasis. We also discussed the clinical research, application, and challenges of LncRNA in TNBC.

## Introduction

Breast cancer (BC) is the most common malignancy currently threatening women’s health globally and is one of the leading causes of cancer death among females, followed by lung carcinoma [1]. It is a highly heterogeneous malignancy that, based on molecular and gene expression analyses, can be characterized into four subtypes: triple negative (basal like), overexpressed human epidermal growth factor receptor 2 (HER2), and ER positive (luminal A and B) [2]. Of these, triple-negative BC (TNBC) represents almost 20% of all BC subtypes and is more common among young females ≤40 years than hormone receptor positive BC [3,4]. TNBC is defined by the absence of ER, progesterone receptor (PR), and HER receptors, biomarkers that can be used to define treatment and to obtain better clinical prognosis. TNBC is characterized by high histological grade, central necrosis, and metastatic potential. Due to the lack of sensitive treatment markers such as ER, PR, and HER2 receptors, TNBC management involves only standard chemotherapy and radiation therapy and results in poor prognosis [5]. In this scenario, in order to transform TNBC from clinically malignant disease to a chronic disease, it is crucial to explore novel molecular targets that can determine the phenotype and predict the clinical prognosis of TNBC.

Non-coding RNA (ncRNA) are divided into short ncRNAs and long ncRNAs (lncRNA) based on the nucleotide length. LncRNA is a class of RNA molecules that do not encode proteins with a length of more than 200 nucleotides [6]. With further research, more and more studies have shown that lncRNAs are abnormally expressed in many cancers, such as BC, lung cancer, prostate cancer, bladder cancer, and pancreatic cancer [7,8]. Unlike miRNA, lncRNA research in cancer is still in its infancy, investigations on how these regulate the biological behavior of cancer such as cancer metastasis, especially in BC are ongoing.

It is well known that distant metastasis is the leading cause of death in BC patients, especially in patients with TNBC. The major reason for the poor prognosis of TNBC is the lack of effective endocrine therapy and targeted therapy in the early stages, which makes TNBC tend to metastatic relapse [9,10]. It is well known that the invasiveness of the cells will be further enhanced when epithelial cells transform into mesenchymal cells, epithelial–mesenchymal transition (EMT) is a dynamic transformation process of malignant tumor epithelial cells into mesenchymal cells, which is the first and important step leading to the metastasis and invasion of malignant neoplasm including TNBC [11]. Recently, several studies have revealed that multiple signaling pathways are related to the EMT process in TNBC, such as Notch signaling, Wnt/β-catenin signaling, Hedgehog signaling, HIF-1α signaling, and PI3K/AKT signaling pathways [12]. For this reason, distinctive EMT-related signaling pathways remain the frontier and focus for overcoming TNBC metastasis and improving patient prognosis.

Numerous studies have shown that lncRNA are involved in the process of EMT and promote the occurrence and development of TNBC [13]. With the further improvement of high-throughput technology, such as RNA sequencing, numerous lncRNAs have been implicated in biological functions such as proliferation, invasion, metastasis, and apoptosis. LncRNAs play a significant role in TNBC metastasis [14]. Additional studies have revealed that MALAT1 induces EMT through the PI3K signaling pathway to promote metastasis in TNBC [15]. However, the role of lncRNAs in the molecular mechanisms of distant metastasis regulating TNBC warrant further study, to explore new therapeutic targets and improve patient prognosis**.**

**Figure 1:**
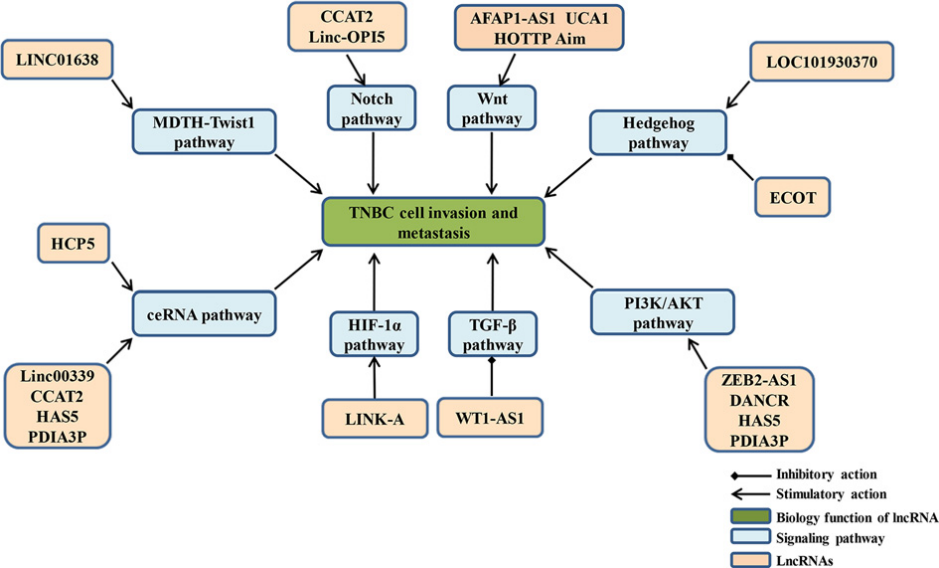
EMT-related signaling pathways mediated by lncRNA regulates the metastasis and invasion of TNBC

**Figure 2:**
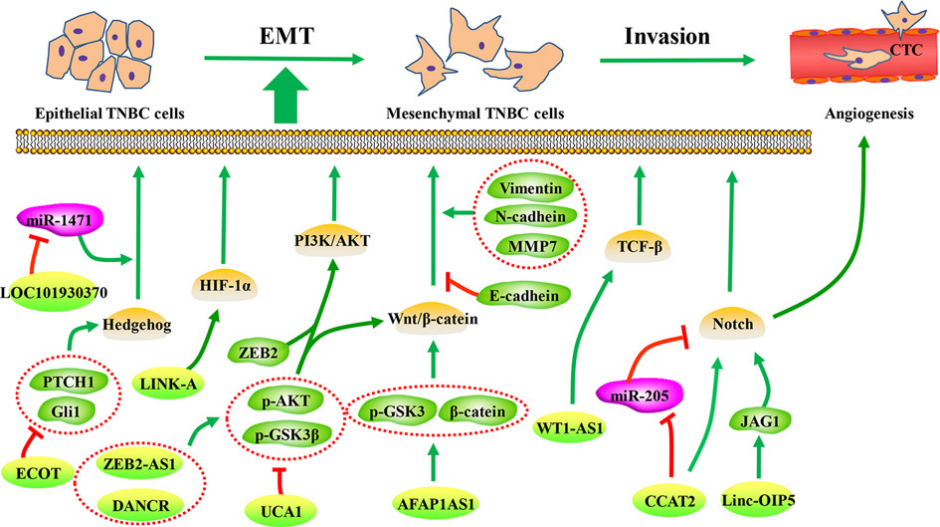
Scheme illustrating validated the metastasis of LncRNA mechanism diagram of EMT-related signaling pathway in TNBC

In this review, we will use the EMT-related signaling pathways involved in TNBC metastasis to organize and summarize the information available relative to the large number of lncRNAs described in the literature, and to form a network diagram of molecular regulatory mechanisms. We will identify many key regulatory lncRNA molecules to explore new therapeutic targets and improve prognosis for TNBC patients (Figures 1 and 2).

## Metastasis-predicament of TNBC

Compared with other subtypes of BC, TNBC patients have a higher probability of recurrence and metastasis, especially to the lung and brain [16]. Mechanistically, TNBC metastasis is strongly associated with the aberrant activation of EMT. EMT is a dynamic complex biological process in which epithelial cells acquire the mesenchymal phenotype, thereby enabling TNBC epithelial cells to acquire strong migration and invasion capabilities, escape from local neoplasia, enter the blood and lymphatic vessels, and eventually colonize in distant organs such as the viscera. From the perspective of molecular markers, the dynamic process of EMT involves the loss of Claudin-1, E-cadherin, and cytokeratin expression, which are replaced by over-expressed vimentin and N-cadherin characterized by the mesenchymal cell phenotype [17]. Moreover, a growing amount of data have shown that there are numerous regulatory factors regulating the EMT process, such as Snail, Slug, ZEB, and Twist. When the function of the tumor suppressor gene P53 is lost or mutated, Snail activity is enhanced indirectly, which in turn promotes the occurrence of BC EMT [18]. Oba et al. reported the microRNAs (miRNA) mir-200c and mir-200b up-regulated the expression of N-cadherin and inhibited the EMT process of BC cells by down-regulating the expression of ZEB2 [19]. Several major signaling pathways also participate in the regulation of the EMT dynamic processes through interaction with regulatory factors, including Notch, Wnt/β-catenin, Hedgehog, HIF-1α, PI3K/AKT, and TGF-β pathways [20]. Leong et al*.* revealed that the Notch signaling pathway was activated after binding to the over-expressed Jagged-1 in BC cells, which further up-regulated the expression of the slug transcription factor and promoted the occurrence of EMT [21]. Therefore, based on the gradual maturity and application of individualized BC treatment, understanding the functions and roles of these factors in the EMT process will provide new effective targets for treatment of TNBC.

## LncRNA in TNBC

The transcriptome of the human genome is very complex. Over 75 of the human genome has the ability to transcribe into transcripts such as ncRNA, while only <2 can be translated into mRNA [22,23]. NcRNA is divided into short ncRNA and lncRNA based on the nucleotide length. LncRNA is a class of RNA molecules with a length of more than 200 nucleotides that do not encode proteins. Based on their genomic location, they can be divided into five categories including antisense lncRNA, sense lncRNA, intergenic lncRNA, intronic lncRNA, and bidirectional lncRNA [24]. However, from the perspective of regulatory mechanisms, lncRNA can be classified into three subtypes: pre-transcriptional regulation, transcriptional regulation, and post-transcriptional regulation [25].

LncRNAs represent a potential therapeutic target and have been identified as biomarkers for various tumors including BC [26]. The current molecular classification of BC is divided into four subtypes based on mRNA expression of ER, PR, and HER2. Unfortunately, their use in TNBC, due to the lack of corresponding molecular markers and the lack of specific treatment options, has led to unsatisfactory clinical outcomes. Thus, it is urgent to find new markers and therapeutic targets for TNBC [27].

Over the past decade, TNBC was divided into four subtypes, including the luminal androgen receptor (LAR) subtype, basal-like and immune suppressed (BLIS) subtype, immunomodulatory (IM) subtype, and mesenchymal-like (MES) subtype according to differences in gene expression, although it seems that the therapeutic effects on TNBC are limited [28]. Recently, improvement in transcriptomic and microarray technologies has revealed that there is a close relationship between numerous lncRNAs and various subtypes of BC [29]. Grembergen et al. used the Gene Expression Omnibus (GEO) database to define the correlation between molecular subtypes and lncRNA expression using the PAM50 test and identified 74 lncRNAs for the basal-like subtype, 14 for HER2 overexpression, 9 for luminal B, and 42 for luminal A BC by detecting lncRNA expression in various subtypes of BC [30]. Further microarray analysis of the transcriptome of 165 TNBC samples revealed that the expression profile of lncRNA differed in each subtype of TNBC. NR_003221, TCONS_00000027 was the highest expressed lncRNA in the MES-subtype and BLIS-subtype TNBC, respectively. ENST00000443397 is a highly expressed lncRNA in the IM-subtype of TNBC, while ENST00000447908 expression is increased in the LAR-subtype of TNBC. Numerous studies have screened specific molecular markers by exploring differences in lncRNA expression between TNBC and non-TNBC tissues. LncRNA BC016831, PRII-434D9.1, and RMST were identified as potential biomarkers of TNBC [31–33].

In recent years, the role of numerous aberrantly expressed LncRNAs in the transfer mechanism has been gradually unveiled in TNBC. Overexpression of GAS5 can promote TNBC progression through competitively down-regulation of miR-196a-5p which is targeted to inhibit the PI3K/AKT signaling pathway. Next, we will summarize the functional roles of lncRNAs involved in TNBC metastasis-related signaling pathways.

## Role of lncRNA-related signaling pathways in triple-negative breast cancer metastasis

The development of novel therapeutic targets and strategies is very important to improve the prognosis of TNBC patients [5]. Ongoing studies have introduced related pathway targeted inhibitors in clinical research [34,35]. Everolimus is a mTOR inhibitor showing strong anticancer activity in preclinical studies. For example, Singh et al. revealed that clinical benefits of metastatic TNBC improve with everolimus and carboplatin [36]. LncRNAs are gradually being identified and are being shown to be specifically expressed in different pathological stages of TNBC, and thus the role of signaling pathway-related LncRNA in TNBC metastasis is gradually being uncovered [37]. Below, we summarize the numerous lncRNAs associated with these promising signaling pathways in order to provide a new frontier for the future treatment of metastatic TNBC (Table 1).

**Table 1:** The clinical significance of signal pathway-related LncRNA in TNBC metastasis

LncRNA	Expression	Acting on signaling pathway	Function
CCAT2	Up	Notch signaling pathway	Overexpressed CCAT2 is significantly associated with poor prognosis of TNBC, CCAT2 affected TNBC cell growth, invasion and metastasis via miR-203/Notch signaling pathway axis.
Linc-OPI5			OIP5 regulated the proangiogenic effect of HUVECs cells through the Notch signaling pathway in the microenvironment of TNBC tissue and thus promoted tumor cell metastasis.
AFAP1-AS1	Up	Wnt/β-catenin signaling pathway	AFAP1-AS1 inhibited MDA-MB-231 cells invsion and migration via β-catenin and p-GSK3. Up-regulation AFAP1-AS1 was correlated with poor OS and DFS in TNBC.
UCA1			Oncogene AFAP1-AS1, HOTTP and Aim affected TNBC metastasis via acting Wnt/β-catenin signaling pathway.
HOTTP			
Aim			
ECOT	Down	Hedgehog signaling pathway	Up-regulated ECOT weaked the migration and invsion of BT549 cell via silenceing PTCH1 and Glil expression.
LOC101930370	Up		LOC101930370 faciliated the biological behavior of breast cancer by regulating miR1417/Hedgehog signaling pathway axis.
ZEB2-AS1	Up	PI3K/AKT signaling pathway	Up-regulated ZEB2-AS1 promoted TNBC metastasis by regulating EMT process.
DANCR			DANCR is highest expressed in TNBC subtype among breast cancer subtypes and associated with TNBC metastasis.
HAS5			HAS5 affected TNBC cell growth and metastasis by sponging miR-196a-5p, as well as PDIA3P sponged miR-183.
PDIA3P			
WT1-AS	Down	TGF-β signaling pathway	Low WT1-AS expression was associated with high clinical grade, overexpression WT1-AS decreased the BT549 cell metastasis through silencing TGF-β1.
LINK-A	Up	HIF-1α signaling pathway	LINK-A was highest expression in TNBC subtype and regulated occurrence and metastasis of TNBC by activating HIF-1α signaling pathway.
LINC01638	Up	MTDH-Twist1 signaling pathway	LINC01638 promoted tumorigenesis of TNBC by preventing SPOP-mediated-c-Myc degradation.
HCP5	Up	ceRNA mechanism	HCP5 inhibited metastasis of TNBC through miR-219a-5p/BIRC3 aixs.
Linc00339			Linc00339 promotes the proliferation, migration, and invasion of TNBC via the miR-377-3P / HOXC6 aixs.

## Notch signaling-related LncRNAs

Notch1 participates in the migration and invasion of breast cancer by regulating the EMT process. Research shows that multiple LncRNAs regulate TNBC metastasis by activating or inhibiting the Notch signaling pathway [38].

The oncogenic lncRNA CCAT2, a colon cancer-related transcript 2, is located on chromosome 8q24.21 with a length of 1752 bp. CCAT2 has been identified to promote proliferation, migration, and invasion in a variety of cancers including colorectal cancer, lung cancer, hepatocellular, and breast cancers, and in particular in TNBC [39]. As an oncogenic lncRNA, overexpression of CCAT2 is significantly associated with poor prognosis. Xu et al. has shown that CCAT2 is more highly expressed in TNBC compared with luminal subtypes of BC. Similarly, metastatic breast cancer cells and breast cancer stem cells (BCSC) are validated. CCAT2 promotes cell invasion and migration of TNBC, while further mechanistic studies have found that CCAT2 regulates TNBC stem cells (TNBCSC) by inhibiting miR-203 expression to activate the Notch signaling pathway [40]. Simultaneously, another study verified that the signaling pathway CCAT2-OCT4-PG1 also mediates TNBCSC regulation through CCAT2. Collectively, the CCAT2-miR-203-Notch and CCAT2-OCT4-PG1 signaling pathways regulate the occurrence and progression of TNBC by regulating the activity of TNBCSC [40].

Linc-OIP5, a novel linc-Opa interacting protein 5, has found to be dysregulated in many malignant tumors [41,42]. For example, Linc-OIP5 as an oncogene promotes biological behaviors such as proliferation, migration, and invasion in lung adenocarcinoma and multiple myeloma. Interestingly, Zhu et al. [43] revealed that linc-OIP5 exerts an important role in MDA-MB-231 cell migration and invasion, mostly through inhibiting JAG1 expression. JAG1, as a key component of the Notch signaling pathway, and contributes to the progression of multiple malignancies [44]. Another study showed that Linc-OIP5 and JAG1 is overexpressed in the TNBC cell line MDA-MB-231 compared with MCF-7 cells. Furthermore, knockdown of linc-OIP5 expression in MDA-MB-231 cells after co-culture with human umbilical vein endothelial cells (HUVECs), the tube-formation and migratory abilities of HUVECs were inhibited. Additionally, JAG1 expression in both the conditioned medium and Notch1 expression on the surface of HUVECs are decreased, which indirectly indicates that the blood vessel formation ability is weakened when expression is down-regulated. It is well known that the formation of new blood vessels is the first step in tumor metastasis. In conclusion, Linc-OIP5 regulates the proangiogenic effect of HUVECs cells through the Notch signaling pathway in the microenvironment of breast cancer and thus promotes TNBC metastasis [45].

## Wnt/β-catenin signaling-related LncRNAs

Wnt/β-catenin signaling pathway is a very complex signal pathway that plays an important role in cell migration, EMT, and stem cell adhesion [46,47]. The Wnt/β-catenin signaling pathway is activated in two ways, named the canonical and noncanonical. The location of β-catenin is the trigger in the canonical pathway, which plays a very vital role in the process of TNBC [48]. Increasing evidence suggests that LncRNA regulates the initiation and progression of BC through the canonical pathway, especially TNBC. Next, we will review the role of lncRNAs associated with Wnt/β-catenin signaling pathway in TNBC metastasis.

The lncRNA AFAP1-AS1, or actin filament-associated protein1-antisense RNA 1, is highly overexpressed in TNBC tissue and cells [49]. Further research has unearthed that higher expression of AFAP1-AS1 was associated with poorer prognosis in TNBC patients including overall survival (OS) and disease-free survival (DFS). Functionally and mechanically, down-regulated AFAP1-AS1 expression significantly inhibited the migration and invasion of MDA-MB-231 cells. In addition, AFAP1-AS1 knockdown also decreased the expression of Wnt/β-catenin signaling pathway-related molecules including β-catenin and p-GSK3. Changes in other EMT-related molecules were observed simultaneously when AFAP1-AS1 was silenced; for example, N-cadherin and vimentin expression were down-regulated, while E-cadherin expression was elevated. Collectively, overexpression of AFAP1-AS1 promotes the dynamic process of EMT through the Wnt/β-catenin signaling pathway to regulate TNBC metastasis and prognosis.

Other lncRNAs are related to the Wnt/β-catenin signaling pathway in TNBC [50–52]. UCA1 was firstly reported in progression of urothelial carcinoma. Recent studies have revealed that UCA1 acts as an oncogene in TNBC. Xiao et al*.* demonstrated that the biological behavior of MDA-MB-231 cells is attenuated after down-regulation of UCA1. Mechanically, p-GSK-3β and GSK-3β, which are negative regulators of the Wnt/β-catenin signaling pathway, were elevated while UCA1 expression was silenced. Besides, β-catenin, Cyclin D1, and MMP-7 expression were decreased. Obviously, UCA1 regulates TNBC invasion and metastasis via the Wnt/β-catenin signaling pathway [52]. With the widespread application of transcriptomics, numerous lncRNAs have been discovered and play important roles in TNBC. LncRNA Aim and HOTTP, as oncogenes, also play an important role in TNBC via activating or silencing the Wnt/β-catenin signaling pathway [50,51]**.**

## LncRNAs related to Hedgehog signaling

The Hedgehog signaling pathway promotes epithelial–stroma interactions through paracrine signaling to regulate the pathogenesis of cancer including TNBC. Recently, Previous studies have shown that the Hedgehog signaling pathway is closely associated with aggressive behavior of breast cancer and the resistance to chemotherapy drugs [53,54]. Xing et al. revealed that the lncRNA BCAR4 as a trigger plays an important role in the metastatic process of TNBC. Further research indicates that BCAR4 is significantly involved in the activation of chemokine-dependent Hedgehog target genes, suggesting the potential role of BCAR4 is to activate the Hedgehog signaling pathway to regulate the epigenetic behavior of organisms [55].

LncRNA ECOT is the abbreviation for the Eosinophil Granule Transcript, which is poorly expressed in BC compared to adjacent tissues. After up-regulation of EGOT expression, the migration and invasion of the TNBC cell line BT549 was weakened. PTCH1 and Gli1, related molecules of the Hedgehog signaling pathway, are silenced when EGOT expression is up-regulated. This phenomenon indicated that EGOT was acting as an oncogene to promote the migration and invasion of TNBC via the Hedgehog signaling pathway [56]. In addition, the same mechanism exists in other subtypes of BC. For example, LOC101930370 regulates the Hedgehog signaling pathway by sponging miR-1417 in breast cancer. However, Hedgehog signaling-related lncRNAs are rarely studied in TNBC [57]. In short, greater attention should be paid to exploring the role of Hedgehog signaling-related lncRNAs in the development and progression of TNBC.

## PI3K/AKT signaling-related LncRNAs

PI3K/AKT signaling pathway activation acts as a trigger in cell proliferation and metabolism in TNBC. PI3K, a phosphoinositide 3-kinase, is an important component of this pathway which is abnormally expressed in many cancers. The PI3K signaling pathway activates AKT/mTOR kinase through a series of complex processes, which affect the biological behavior of tumors [58]. Several PI3K/AKT signaling pathway inhibitors, such as selective PI3K and Akt inhibitors, are currently being tested in clinical trials for TNBC. Everolimus, an mTOR inhibitor, has been approved for the clinical treatment of breast cancer, but is still in phase 1 clinical trials in TNBC [58,59]. Recent studies have shown that numerous lncRNAs also act on TNBC processes by regulating the PI3K/AKT signaling pathway. Herein, we list the lncRNAs involved in this pathway.

ZEB2-AS1 functions as an oncogene in TNBC. ZEB2-AS1 is markedly up-regulated in TNBC tissues and is associated with lymph node metastasis. Down-regulation of ZEB-AS1 in MDA-MB-231 cells inhibited cell proliferation and invasion. Similarly, ZEB-AS1 attenuated tumor proliferation and lung metastasis in SCID mice. ZEB2, a major protein in the ZEB family, is a crucial transcription factor that regulates the EMT process in numerous malignancies, and is down-regulated after knocking down ZEB-AS1 expression. Subsequently, GSK3β and AKT phosphorylation levels are suppressed when ZEB-AS1 expression is down-regulated. Overall, lncRNA ZEB-AS1 promotes proliferation, metastasis, and EMT by activating the PI3K/AKT signaling pathway in TNBC [60].

Differentiation Antagonizing Non-protein Coding RNA (DANCR), located on human chromosome 4q12, acts as an oncogene in multiple cancers including TNBC [61,62]. DANCR is highly expressed in TNBC subtypes compared with HER2 and luminal BC subtypes. Patients with high expression of DANCR have a worse prognosis. DANCR promotes phosphorylation of RXRA depending on GSK3β, leading to the activation of the PI3K/AKT signaling pathway and ultimately promotes tumorigenesis of TNBC [63]. With the application of high-throughput technology, increasing lncRNAs have been found to regulate TNBC tumorigenesis through the PI3K/AKT signaling pathway. Numerous studies have demonstrated that lncRNA acts as a competitive endogenous RNA (ceRNA), which activates the PI3K/AKT signaling pathway through sponge miRNA to regulate the tumorigenesis of TNBC [64,65]. For example, lncRNA HAS5 competitively binds to miR-196a-5p and lncRNA PDIA3P competitively binds to miR-183, both of which activate the PI3K/AKT signaling pathway to promote the invasion and metastasis of TNBC.

## TGF-β signaling pathway-related lncRNAs

Studies have demonstrated that transforming growth factor β (TGFβ) is associated with the EMT, which is involved in carcinogenesis [66]. TGFβ, one of three subtypes of TGFβ (TGFβ1, TGFβ2, and TGFβ3), is the most widely studied cytokine in BC. In a transgenic mouse model of breast cancer, overexpression of TGF-β1 continued to induce activation of the TGF-β signaling pathway, increasing the potential of tumor cells to direct lung metastasis [67]. In contrast, lncRNA related to the TGF-β signaling pathway is less studied in TNBC. Recently, some studies have revealed that lncRNA acts as a trigger in the activation of the TGF-β signaling pathway in the carcinogenesis of TNBC.

Numerous studies have shown that the low expression of WT1-AS plays a critical role in promoting tumorigenesis in many malignancies including cervical cancer and gastric cancer [68,69]. Recently, Wang et al*.* demonstrated that the expression of lncRNA WT1-AS was markedly reduced in TBNC tissues and in the BT459 cell line. Surprisingly, WT1-AS1 expression is inversely associated with clinical grade, indicating that lower WT1-AS1 expression has a worse prognosis for patients. Overexpression of WT1-AS1 leads to a decrease in the migration and invasion ability of TNBC cells BT549, and simultaneously the expression of TGF-β1 is also weakened. Furthermore, up-regulated TGF-β1 can rescue the siRNA WT1-AS1, which leads to a decrease in the migration capacity. The study concluded that WT1-AS regulates tumorigenesis of TNBC through the TGF-β signaling pathway [70]. However, additional research is needed to study the lncRNA associated with the TGF-β signaling pathway in order to identify potential molecular markers suitable for clinical application to improve the prognosis of patients with TNBC.

## Other signaling pathway-related lncRNAs

Emerging evidence has suggested that ceRNA is a type of ncRNA that binds to microRNA (miRNA) and plays a critical role in the pathophysiological process of the organism. LncRNA antagonizes the inhibitory effect of miRNA on coding genes by combining with miRNA response elements (MREs) [7,22]. Wang et al. have reported that the ceRNA lncRNA HCP5 can regulate BIRC3 expression through competitively sponging miR-219a-5p in the progression of TNBC [71]. BIRC3 is an oncogene and apoptosis-inhibiting gene in many cancers, and has been shown to promote the metastasis of TNBC [72]. Han et al*.* identified a lncRNA CCAT1/miR-218/ZFX signaling pathway that regulates TNBC progression. CCAT1 expression is up-regulated in TNBC tissues and cells. Silencing CCAT1 results in increased miRNA-218 expression and then up-regulated ZFX expression. CCAT1 promotes TNBC invasion and metastasis through miRNA-218/ZAF axis signaling pathway [73]. Linc00339, a recently identified LncRNA found to regulate the biological behavior of many tumors. Wang et al*.* showed that overexpression of Linc00339 promotes the proliferation, migration, and invasion of TNBC through the miR-377-3P/HOXC6 signaling pathway [74].

Other signaling pathway-related lncRNAs also play important roles in the tumorigenesis of TNBC, through hypoxia-inducible factor (HIF)-1α signaling and other signaling pathways [75,76]. HIFs promote metastasis, relapse, and metabolic reprogramming in TNBC. LncRNA LINK-A, or Long-Intergenic Noncoding RNA For Kinase Activation, is highly expressed in TNBC tissues compared with the luminal and HER2 subtypes. Mechanically, LncRNA LINK-A regulates the onset and development of triple-negative BC by activating the HIF-1α signaling pathway [77]. Higher LncRNA LINK-A expression is associated with a worse prognosis. Another over-expressed LncRNA, LINC01638, is a risk factor for poor prognosis in TNBC [78]. Knockdown of LINC01638 can inhibit tumor proliferation and metastasis *in vivo* and *in vitro*. Further research determined that LINC01638 regulates the biological behavior of TNBC by activating the MTDH-Twist1 signaling pathway which prevents SPOP-mediated c-Myc degradation.

## Clinically targeted RNA therapy

TNBC lacks targeted and endocrine therapies similar to those that can be applied to HER-2 and luminal BC subtypes, making TNBC very aggressive and having a poor prognosis [9]. Numerous lncRNAs are specifically expressed in TNBC tissues and cells, and are significantly related to the prognosis of TNBC patients [27]. The study of lncRNAs related to different signaling pathways *in vivo* and *in vitro* has provided incredible targets for the treatment of TNBC (Figure 1), although this research area is still in its infancy. Herein, we will introduce the current state of research in this field.

Some studies have used targeted knockout specific gene technology such as small interfering RNAs (siRNA) and antisense oligonucleotides (ASOs) in clinical stage I and II BC [79]. However, currently in clinical trials the target genes of such siRNA and ASOs are mainly mRNA, and not LncRNA. For example, the siRNA drug siG12D LODER and conventional chemotherapeutics are being used in combination to treat malignancies [80]. Recently, nanoparticles (NPs) have been used as more efficient carriers for siRNA, ASOs, and chemotherapeutics due to their safety profile, fewer adverse immune reactions, and strong cell type targeting [81]. This will provide new hope for the treatment of metastatic TNBC [82]. For example, CDK1 siRNA conjugated with the cationic lipid-assisted PEG-PLA NPs carrier and siRNA targeting anti-CD44 aptamer with protamine-conjugated NPs, effectively weakens the biological function of TNBC [83,84]. This biotechnology involving siRNA-conjugated NPs will pave the way for targeted treatment of advanced TNBC through targeted delivery of siRNA. What is exciting is that Schiemann et al*.* have efficiently delivered siRNA (LncRNA DANCR) to target cells through nanoparticle-mediated RNAi (RGD-PEG-ECO/siDANCR NPs). *In vitro* experiments show that RGD-PEG-ECO/siDANCR NPs are more effective in silencing DANCR, resulting in significantly reduced proliferation, migration, and invasion by TNBC cells MDA-MB-231 and BT549. Furthermore, NPs have been used to treat TNBC xenograft-bearing nude mice by strongly inhibiting the progression of TNBC tumors [85]. In the future, combined delivery to TNBC cells using NPs—carrying a combination of inhibitors of signaling-related LncRNA and inhibitors of signaling pathways [82]—will result in high efficiency synergistic effects of drugs. These results and hypotheses suggest that nanoparticle-mediated siLncRNA will provide effective, accurate, and personalized treatment options for TNBC patients in the future.

## Future prospects and challenges

In conclusion, mRNA and miRNA as potential molecular biomarkers have made significant progress for early clinical detection and as therapeutic targets of specific tumors, but lncRNA exploration in cancer is still holds much to discovers [26]. Nonetheless, emerging evidence has demonstrated that lncRNAs play a key role in the tumorigenesis and development of various tumors including BC, especially TNBC which has aggressive biological behavior [7]. With the application of transcriptomics and high-throughput technology, a large amount of data indicates that the differential expression of lncRNA is significantly related to BC subtypes [86]. For example, TNBC subtype-related lncRNAs include MEG3, DANCR, and MALAT1. It is well known that TNBC patients are in dire need of effective targeted drugs and endocrine drugs, which makes the survival rate and disease-free survival of patients with distant metastasis not optimistic [58]. The identification of specific molecular markers, such as lncRNA, related to metastasis for the treatment of TNBC patients will result in significant progress.

In this review, we briefly introduced the dilemma of TNBC treatment and propose strategies on how to overcome it. We have summarized the numerous signaling pathways involved in TNBC metastasis and the lncRNAs related to these signaling pathways. We found that lncRNA regulates the EMT process by affecting the activity of these signaling pathways to promote the metastasis of TNBC (Figure 2). Depending on screening signaling pathways regulated by lncRNAs, we can use signaling pathway-related inhibitors and specific siRNA (targeting signaling pathway-related lncRNA) to verify whether they will produce synergistic effects. Nonetheless, the challenge remains on how to target the lncRNA to cancer cells. NPs are a type of delivery vehicle with limited side effects, but strong targeting capacities and high efficiency. Some studies have explored the efficacy of drug delivery via NPs in breast cancer. For example, Schiemann et al*.* efficiently delivered siRNA targeting lncRNA DANCR to TNBC cells using NPs-mediated RNAi (RGD-PEG-ECO/siDANCR NPs). However, NPS are still in the preliminary stages of development as delivery vehicles, and many difficulties still need to be overcome. Therefore, the combination of metastasis associated LncRNA and NP carriers provides very promising anti-TNBC therapy. Further insight into the NP delivery system, technical innovation, and exploration of significant metastasis-related lncRNAs will greatly benefit potential molecular targeted therapies for TNBC patients, thereby further improving disease-free survival and quality of life for patients with metastatic TNBC.

## Abbreviations

ASO: antisense oligonucleotide
DFS: disease-free survival
EMT: epithelial–mesenchymal transition
HER2: human epidermal growth factor receptor 2
HUVEC: human umbilical vein endothelial cell
LncRNA: long non-coding RNA
OS: overall survival
TGFβ: transforming growth factor β
TNBC: triple-negative breast cancer
